# The prevalence of prolonged grief disorder in patients with common mental disorders: a comparative study between patients with and without a refugee background in the Netherlands

**DOI:** 10.3389/fpsyt.2026.1809816

**Published:** 2026-07-29

**Authors:** Simon P. N. Groen, Daniëlle C. Cath, Rob H. S. van den Brink, Geert E. Smid

**Affiliations:** 1 De Evenaar Center for Transcultural Psychiatry Drenthe Mental Health Care, Beilen, Netherlands; 2 Research Department Common Mental Disorders, GGZ Drenthe, Assen, Netherlands; 3 Department of Psychiatry, University Medical Center Groningen, Groningen University, Groningen, Netherlands; 4 Department of Humanist Chaplaincy Studies, University of Humanistic Studies, Utrecht, Netherlands; 5 ARQ National Psychotrauma Centre, Diemen, Netherlands

**Keywords:** anxiety, common mental disorders, co-occurrence, depression, PGD, posttraumatic stress, prevalence, prolonged grief disorder

## Abstract

**Background:**

The recent inclusion of prolonged grief disorder (PGD) in international classification systems marks worldwide recognition of the potential impact of bereavement on mental health. PGD is often overlooked in psychiatric care, and little is known about the prevalence of PGD among individuals referred to secondary mental health care for treatment of common mental disorders including anxiety disorders, posttraumatic stress disorder (PTSD), and depression. Symptoms of these common mental disorders may overlap with PGD symptoms.

**Objective:**

In the current pilot study, we investigated the prevalence of PGD in N = 45 patients with common mental disorders referred to outpatient clinics of a secondary mental health care center who reported having lost a loved one due to death.

**Methods:**

We included patients with (n=19) and without (n=26) a refugee background, to compare individuals who lost a loved one in different contexts. The Traumatic Grief Inventory-Clinician Administered (TGI-CA) was used to assess the prevalence of PGD and PGD symptom severity and the Mini-SCAN to diagnose common mental disorders.

**Results:**

Up to 34% of the sample had a PGD according to both DSM-5-TR and ICD-11 criteria, against 9,8% in general populations. The TGI-CA scoring methods showed perfect agreement between ICD-11 and DSM-5-TR criteria. In univariate analyses, refugees had a two to three times higher risk of PGD compared to non-refugees, and higher PGD symptom severity was associated with being female, refugee, and having an anxiety disorder or PTSD. In multivariate analysis, the influence of refugee status on PGD symptom severity proved to be most prominent for younger patients.

**Conclusions:**

These pilot study results suggest that it is recommendable to investigate PGD in larger samples and to assess the TGI-CA among patients with common mental disorders, particularly in refugee patients.

## Introduction

The loss of a loved one may lead to prolonged grief reactions that have a potential impact on mental well-being and daily functioning, especially when the loss is experienced as unexpected and sudden. Feelings of intense emotional pain, disbelief about the death of the loved one, intense loneliness, and disruption in identity that persist over time may lead to mental health problems that require treatment (1). Formal recognition of such mental health problems is established as Prolonged Grief Disorder (PGD) in the fifth edition, text revision, of the Diagnostic and Statistical Manual (DSM-5-TR, 2) and the eleventh edition of the International Classification of Disorders (ICD-11, 3). Meta-analysis has resulted in a pooled prevalence estimate of 9,8% PGD (95% CI 6.8–14.0) in non-psychiatric adult populations exposed to non-violent bereavement (4). A recent cross-national analysis showed an average PGD prevalence of 13% varying from 5% to 16% in non-probability samples in sixteen countries with over 20,000 participants (5). When individuals are exposed to loss as a result of a sudden and violent death of a loved one, pooled prevalence rates of probable PGD increase to 49% (95% CI 33.6-65.4) (6). These prevalence rates need to be considered with some caution (7). More recent studies in Germany showed pooled prevalence rates from 3.3% to 4,7% (DMS-5-TR) and 4,2% to 5,4% PGD (ICD-11) in bereaved individuals (8, 9).

Some symptoms of PGD resemble or overlap with symptoms of common mental disorders, including anxiety disorders, depression, and posttraumatic stress disorder (PTSD). Symptoms that overlap between PGD and depression are sorrow, loss of interest, feelings of worthlessness, and feelings of guilt. Symptoms shared by PGD and PTSD are feelings of shock, intrusive memories, and avoidance of situations that provoke memories of traumatic events. Overlap with these common mental disorders poses a challenge for the recognition of PGD in clinical practice as a distinct disorder in bereaved individuals. A metareview of 23 studies in bereaved adults shows pooled prevalence estimates of PGD symptoms with co-occurrent depression (63%), anxiety (54%) and posttraumatic stress symptoms (49%; 10). A latent class analysis of PGD, PTSD and major depression disorder in a Dutch sample of 496 bereaved individuals showed three classes: the largest class with PGD (48%), a combined PGD/PTSD class (27%) and a resilient class with neither PGD nor PTSD (25%; 11). Depression severity did not differ between these classes. These studies show that specific symptom patterns emerge after exposure to trauma and loss. An epidemiological study the United States showed that one in two individuals experienced unexpected loss, which was related to the onset of depression, panic disorder, manic episodes, alcohol use, and PTSD (12).

In particular, individuals in war and (post-)conflict areas seem to run a high risk of losing a loved one as a result of sudden and violent deaths. In case individuals seek refuge, they may also be confronted with bereavement during and after migration. In a Dutch study in Iraqi asylum seekers, 87,6% lost a loved one (13) and in a German sample of asylum seekers, 92% lost a loved one (14). The aforementioned cross-national analysis showed that PGD prevalence rates substantially differed between representative and convenience samples (5% vs. 16%), depending on country characteristics such as variation in adverse effects of disasters, and in mean age of the population studied, with older mean age being associated with higher PGD rates (5). This study, however, included primarily European and North-American samples, and not specifically war, conflict or post-conflict areas. The only study that directly compared individuals from non-conflict and conflict areas, including n=73 French and n=162 Togolese widowed individuals, showed no between-group differences in PGD prevalence rates, but etiology, risk and protective factors were considered culturally distinct (15).

Co-occurrence of PGD with symptoms of common mental disorders is frequently reported (10). Latent class analysis in 248 refugees who were exposed to trauma and loss and who completed interviews assessing symptoms of PGD and PTSD resulted in four classes: a predominantly PGD class (16%), a combined PGD/PTSD class (16%), a predominantly PTSD class (25%), and the largest class being a resilient class (43%; 16). In the PGD class of the refugee sample, postmigration adaptation difficulties after relocation were frequent, whereas refugees in the PTSD class were more likely to have experienced difficulties related to loss of culture and support.

In the present study, we first aim to examine the prevalence of PGD and determinants of PGD and PGD symptom severity in a small sample of refugee and a non-refugee patients referred to outpatient clinics in secondary mental health care, who had lost a loved one at least one year prior to mental health treatment. Second, we aim to assess co-occurrence of PGD and associations of PGD symptom severity with diagnoses of common mental disorders. We hypothesize that the prevalence of PGD symptoms in refugees is higher than in non-refugees. Further, we hypothesize that among common mental disorders PTSD is most co-occurrent with PGD and occurs more frequently in refugees than in non-refugees.

## Methods

### Study population and procedure

Data collection took place from December 2021 to June 2023 at three outpatient clinics of a mental health care organization in the northern part of the Netherlands. Inclusion criteria were a minimum age of 18 years, loss of a loved one due to death after the age of five and more than one year preceding referral, symptoms of anxiety, depression or PTSD as primary reason for referral, and being able to fill out self-report questionnaires. Interpreter assistance was provided when needed. New patients were recruited in the period of initial psychiatric assessment. During the recruitment period, 168 consecutive patients were found eligible for the study. A number of patients could not be included, because they did not continue the treatment process, in other cases communication between clinicians and researchers did not lead to inclusion After being informed about the study, 62 patients willing to participate remained. Of those patients, sixteen were excluded or refused to participate after being informed about the study. Main reasons for refusing participation were: patient prioritizing trauma treatment above study participation (n=5), fear that talking about the loved one would evoke strong emotions (n=4), feeling unable to talk about the deceased loved one (n=4), and stopping of treatment (n=3). We included 46 patients of whom 27 (59%) had a refugee background.

### Measures

The Traumatic Grief Inventory-Clinician Administered (TGI-CA) was employed to assess PGD symptom severity and diagnosis of PGD. This 22-item version with a 5-point Likert scale (never-rarely-sometimes-frequently-always) used by clinicians is considered to be more reliable than the self-report version (17). The items cover the symptoms of a PGD diagnose according to both the DSM-5-TR and ICD-11 (see below). The sum score of the 22 items assesses PGD symptom severity. Psychometric properties of this score are good (18, 19). The Cronbach’s α measure for the internal consistency of the 22 items was 0.92 in the present study. The TGI-CA is also used to diagnose PGD according to criteria in DSM-5-TR (4) and ICD-11 (3, 20). For the ICD-11 two different sets of criteria for a PGD diagnoses have been developed; a ‘liberal’ and a ‘conservative’ set (21). We used the ‘liberal’ set. Self-response versions of the Traumatic Grief Inventory have been translated in several languages and applied in several cultural contexts (e.g., 22–24).

To assess diagnoses of anxiety, depression and posttraumatic stress disorder according to DSM-5, we employed the Mini-SCAN, which is a semi-structured psychiatric interview (25). The Mini-SCAN is a shorter version of the Schedules for Clinical Assessment in Neuropsychiatry (SCAN) of the World Health Organization (26). The Mini-SCAN shows good validity with DSM-5 diagnoses, with most kappa values around 0.90 (27).

### Data analysis

We first examined the demographic and clinical characteristics of the refugee and non-refugee participants, including PGD prevalence according to ICD-11 and DSM-5-TR definitions, and tested for differences between the two groups (by Student’s t tests for continuous measures and Chi-square tests for categorical ones). We then studied determinants of (1) PGD diagnosis and (2) PGD symptom severity and tested whether these determinants were different for the refugee and non-refugee groups (by testing for interactions of the determinants and the grouping variable). Determinants of PGD diagnosis were examined using logistic regression analysis, in which the strength of the relationship is expressed as Odds Ratio (OR) for consecutive levels of the determinant with a 95% Confidence Interval (95% CI). Determinants of PGD symptom severity were examined using linear regression analysis, with the TGI-CA total score as dependent variable. The regression coefficient B (and its 95% CI) indicates the expected difference in mean TGI-CA total score between consecutive levels of the determinant. Both regression analyses were first conducted univariately, to examine the relationships of individual determinants with PGD and PGD symptom severity, and then multivariately, to examine the mutually correlated influences of the combined determinants. For both regression analyses, entrance of determinants in the multivariate analyses was block wise, with the demographic variables (including refugee status) entered first, and the common mental disorders diagnoses entered second. This was done to specifically examine the added value of the diagnostic information to the demographic information in predicting PGD diagnosis or symptom severity in bereaved patients referred for a common mental disorder.

## Results

**Table 1** shows the characteristics of the study sample. Mean age of the total sample was 42.2 years (SD = 12.1) and 40% were female. Countries of origin of the refugees were Bosnia (1), Eritrea (2), Iran (6), Iraq (2), Nigeria (2), Somalia (1), Syria (7), Turkey (1), Yemen (1), and four individuals’ country of origin was unknown. According to the Mini-Scan, three participants neither met criteria for anxiety, depression nor PTSD diagnosis. With respect to the different definitions of PGD diagnosis studied, the definitions according to DSM5-TR and ICD-11 showed perfect agreement in the present sample, which means that exactly the same persons did or did not have a PGD according to both definitions. The analyses using these definitions of PGD were therefore combined. Criteria of a PGD diagnosis were fulfilled by 46% of the refugees, and 17% of the non-refugees (χ^2^ = 4.12; df=1; p=.042), and PGD symptom severity was significantly higher in the refugees than the non-refugees. In addition, the refugees were somewhat younger than the non-refugees, and considerably more often had a PTSD diagnosis (73% versus 21%; t=11.89; df=1; p<.001).

**Table 1:** Characteristics of the study sample.

Characteristic	Total sample (*n*=46)	Refugees (*n*=27)	Non-refugees (*n*=19)	Difference	
				t/χ^2^	p
Age, mean (SD)^a^	42.2 (12.1)	38.9 (10.2)	47.2 (13.3)	2.36	.023
Female, n (%) ^a^	18 (40%)	9 (33%)	9 (50%)	1.25	.264
Prolonged Grief Disorder DSM5, n (%)^b^*	15 (34%)	12 (46%)	3 (17%)	4.12	.042
Prolonged Grief Disorder ICD11, n (%) ^b^ *	15 (34%)	12 (46%)	3 (17%)	4.12	.042
Prolonged Grief Disorder symptom severity, mean (SD)	65.2 (18.1)	70.3 (16.3)	58.1 (18.5)	2.36	.023
Posttraumatic stress disorder, n (%) ^a^	23 (51%)	19 (73%)	4 (21%)	11.89	<.001
Depressive disorder, n (%) ^a^	40 (89%)	24 (92%)	16 (84%)	0.73	.393
Anxiety disorder, n (%)^c^	28 (65%)	18 (75%)	10 (53%)	2.34	.126

Number of missing responses: ^a^n=1; ^b^n=2; ^c^n=3.

*Perfect fit between the two classifications.

Study participants had lost a loved one due to death in various ways. Most participants had lost one parent, either father or mother. Some participants lost a partner, some a child, a friend, a grandparent or another relative. A minority experienced multiple losses, especially among refugees. The circumstances of death varied from natural death, to death due to illness, to death because of violence. Especially among refugees, many experienced war-related deaths. Some deaths were related to migration due to cancer of a participant who used to take care of her. Some grief processes had culturally specific elements, such as the death of a mother of a lesbian young woman who lost her main source or protection in a country that forbids homosexuality.

**Table 2** shows the results of the logistic regression analyses of determinants of PGD diagnosis. Interestingly, only refugee status was related to the presence of a PGD diagnosis. None of the other determinants contributed to the prediction of PGD (see **Table 2**), and none showed a significant interaction with refugee-status (all p>0.10), which means that there was no evidence that any of the determinants had additional predictive value for PGD diagnosis in the refugees compared to the non-refugees.

**Table 2:** Determinants of Prolonged Grief Disorder^a^.

Determinant	Univariate		Multivariate			
			*Block 1 entered^b^*		*Blocks 1 & 2 entered^b^*	
	OR (95%CI)	p	OR (95%CI)	p	OR (95%CI)	p
Age	0.96 (0.90; 1.02)	.14	0.97 (0.91; 1.03)	.32	0.96 (0.89; 1.03)	.26
Sex (female)	1.58 (0.44; 5.64)	.49	2.25 (0.54; 9.28)	.26	2.21 (0.48; 10.06)	.31
Refugee	4.29 (1.00; 18.45)	.05	3.51 (0.71; 17.27)	.12	5.33 (0.66; 42.94)	.12
Posttraumatic stress disorder	1.22 (0.35; 4.27)	.75			0.30 (0.04; 2.22)	.24
Anxiety disorder	2.35 (0.53; 10.41)	.26			1.49 (0.24; 9.12)	.67
Depressive disorder	2.24 (0.23; 22.05)	.49			1.23 (0.10; 14.66)	.87

^a^ According to both DSM-5 and ICD-11 criteria.

^b^ Block 1 consisted of the determinants age, sex, and refugee, block 2 of posttraumatic stress disorder, anxiety disorder, and depressive disorder.

**Table 3** shows the results of the linear regression analyses of determinants of PGD symptom severity. Refugee status and age had a significant interaction effect on PGD symptom severity (F = 5.36, p=.03). For example, in the youngest patients included, aged 18 years, a very large effect of refugee status was found on PGD symptom severity (B = 41.15; 95%CI: 16.81; 65.50; t=3.44, p<.01 on the TGI-CA total score; controlled for all other determinants studied), but this effect decreased with 1.21 (0.40; 2.02) points per year increase in age, so that the effect in, for example, 50 year old patients was reduced to 2.49 (-9.29; 14.27) and was no longer significant (t=0.43; p=.67). The interaction effect between age and refugee status can also be seen from the fact that PGD symptom severity was not significantly related to age in non-refugees (B = 0.38; 95%CI: -0.15; 0.92; t=.1.47; p=.15; again after controlling for all other determinants), but significantly decreased with 0.82 (0.21; 1.44; t=.2.71; p=.01) points per year in the refugees. In addition, female sex and having an anxiety disorder were related to PGD symptom severity. Both females and patients with an anxiety disorder scored higher on the TGI-CA (approximately 15.34 and 12.36 points, respectively), independently of the other determinants studied. No independent effects were found for a posttraumatic stress or depressive disorder.

**Table 3:** Determinants of Prolonged Grief Disorder symptom severity.

Determinant	Univariate			Multivariate					
				*Block 1 entered^a^*			*Blocks 1 & 2 entered^a^*		
	B (95%CI)	t	p	B (95%CI)	t	p	B (95%CI)	t	p
Age^b^	-0.38 (-0.83; 0.07)	1.71	.10	0.29 (-0.26; 0.83)^d^	1.06	.29	0.38 (-0.15; 0.92)^f^	1.47	.15
Sex (female)	11.28 (0.55; 22.01)	2.12	.04	15.83 (6.57; 25.08)	3.46	<.01	15.34 (5.93; 24.75)	3.31	<.01
Refugee	12.15 (1.77; 22.54)	2.36	.02	41.85 (19.69; 64.01)^e^	3.82	<.01	41.15 (16.81; 65.50)^g^	3.44	<.01
Age^b^ X Refugee	NA^c^			-1.19 (-1.99; -0.40)	3.04	<.01	-1.21 (-2.02; -0.40)	3.04	<.01
Posttraumatic stress disorder	10.61 (0.00; 21.22)	2.02	.05				-3.79 (-15.41; 7.83)	0.66	.51
Anxiety disorder	18.44 (7.90; 28.98)	3.53	<.01				12.36 (1.70; 23.03)	2.36	.02
Depressive disorder	12.98 (-4.23; 30.18)	1.52	.14				6.98 (-7.36; 21.33)	0.99	.33

^a^ Block 1 consisted of the determinants age, sex, refugee, and the interaction between age and refugee, block 2 of posttraumatic stress disorder, anxiety disorder, and depressive disorder.

^b^ Age minus 18 years.

^c^ Not applicable.

^d^ Effect of age in non-refugees controlled for other block 1 variables is shown in table. Effect in refugees is -0.91 per year (-1.48; -0.33), t=3.18, p<.01.

^e^ Effect of refugee status controlled for other block 1 variables in 18-year-old persons is shown in table. Effect decreases with 1.19 (0.40; 1.99), t=3.04, p<.01 per increasing year of age, and is, for example, for 50-year-old persons reduced to 3.65 (-7.50; 14.79), t=0.66, p=.51.

^f^ Effect of age in non-refugees controlled for block 1 and 2 variables is shown in table. Effect in refugees is -0.82 per year (-1.44; -0.21), t=2.71, p=.01.

^g^ Effect of refugee status controlled for block 1 and 2 variables in 18-year-old persons is shown in table. Effect decreases with 1.21 (0.40; 2.02), t=3.04, p<.01 per increasing year of age, and is, for example, for 50-year-old persons reduced to 2.49 (-9.29; 14.27), t=0.43, p=.67.

## Discussion

To the best of our knowledge, this is the first study that has investigated rates of PGD in patients referred with common mental disorders to outpatient clinics in secondary mental health care, comparing those with versus without a refugee background (28). We examined the prevalence of PGD in 46 refugee and non-refugee patients referred for treatment of common mental disorders who lost a loved one due to death at least twelve months prior to referral. The results show that approximately one third of the sample (34%) met ICD-11 and DSM-5-TR criteria for PGD using the Traumatic Grief Inventary-Clinician Administered (TGI-CA). This prevalence is considerably higher than the pooled prevalence of 9.8% PGD in non-psychiatric adult populations exposed to non-violent bereavement (4), higher than the 13% cross-national prevalence found when taking country vulnerability into account (5) and higher than the 15.8% probable PGD in a refugee non-psychiatric population in Australia (29), and lower than the 49% pooled prevalence of PGD directly following unnatural and violent loss (6). However, when differences between refugees and non-refugees are considered, the prevalence of PGD in our refugee patient group was 46% according to both DSM-5-TR and ICD-11 scores, which is in line with the prevalence rates found by Djelantik et al. in refugees. Considering the high potential of unnatural and violent loss in refugee populations who migrated to Europe (14, 30), the findings in this study seem consistent with that notion. However, likely as a result of the fact that our group was recruited at outpatient clinics in secondary mental health care, in contrast to the Djelantik et al. sample, individuals additionally suffered from high rates of common mental disorders.

Interestingly, in our study we found no difference between PGD cases identified by the DSM-5-TR and the ‘liberal’ scoring criteria of the ICD-11. Exactly the same participants were identified with a PGD, although the criteria and hence TGI-CA items used to identify the cases differs between both classification systems (20, 31). This finding is in line with a large-scale cross-national analysis, that found no differences between ICD-11 and DSM-5-TR derived scores for PGD prevalence (5). A German study found a variance of 4.7-6.8% PGD TGI-scores in a representative sample of the general population *(n=*2,509), with considerably lower prevalence rates according to DSM-5-TR criteria than ICD-11 criteria (9), which might result from the differences in criteria and TGI scales being developed with a Global North-orientation. The TGI has been designed to both address Persistent Complex Bereavement Disorder (PCBD) in DSM-5 and Prolonged Grief Disorder in ICD-11 (31). Since DSM-5-TR, both disorders are named PGD, but criteria of PGD vary between DSM and ICD. In ICD-11, PGD can be diagnosed from six months onward after bereavement, but PGD according to DSM-5-TR can be diagnosed at twelve months after bereavement. Furthermore, assessing grief in refugee and post conflict populations is inherently related to mostly Global North-based (formerly labeled as Western societies), etic measurements (i.e., using outsider perspectives) instead of using locally developed, emic (i.e., using insider perspectives) and culturally adapted measures, which can lead to over- and underestimation of actual traumatic grief response. A narrative review of ‘etic’ and ‘emic’ approaches showed that the prevalence of PGD could be high depending on the level of cultural adaptation of the individual (32). In this study only etic measures were employed, but no culturally adapted measures.

Our analyses showed that being a refugee but not suffering from a mental disorder was associated with having a PGD diagnosis (**Table 2**). Interestingly, common mental disorders including PTSD, anxiety or depression are most prevalent in refugees (10, 33), which is in line with our study results. A systematic review on prospective longitudinal studies revealed that PGD symptoms more consistently predict symptoms of depression and posttraumatic stress than vice versa, but not anxiety (34). Our study was only partly in line with these findings; although the cross-sectional nature of the current sample does not allow predictive inferences. PGD symptoms were not associated with depressive symptoms but with anxiety disorder and PTSD. Apparently, PGD diagnoses and mental disorders are not linearly associated and run a partly independent course, however, PGD symptoms may contribute to onset and/or maintenance of common mental disorders.

Refugees run a high risk of acquiring a PGD, which is in line with findings of a population study in Australia (29). Studies have already pointed out the high level of sudden and traumatic bereavement in refugees and the current study results underline this, with a two to three times higher chance of probable PGD than in non-refugees. In general, clinicians are less aware of the diagnosis of PGD, and in refugees tend to diagnose PTSD more often when in fact their patients with experiences of traumatic bereavement suffer from PGD which requires a somewhat different psychotherapeutic approach. Therefore, inquiring about the loss of loved ones needs more attention in mental health assessments, particularly of refugees. Not only do refugees have a higher risk of losing loved ones, they also face challenges of adapting to a new society, post-migration living problems and cultural identity confusion (35, 36). These processes may influence the grief process of these refugees. Although international standards are met in the development of instruments to assess PGD, we must take into account that the cross-cultural validity of these instruments may be limited (37).

Female gender was associated with a PGD diagnosis in our study, although we did not directly compare PGD symptoms between men and women. Gender differences in bereavement and grief studies have shown mixed results in the literature. Some studies acknowledge gender differences in grief reactions (38, 39), others found no gender differences (40). Underlying characteristics of grief reactions in men versus women is rarely studied and poorly understood. 39, found that widowers who experienced the loss of a loved one three years or longer ago, showed increased bitterness, whereas widows were found to have lower levels of complicated grief (39). In addition, women may have a more avoidant coping style than men following the death of a parent (40). In contrast, a network analysis among older individuals who lost their spouse resulted in more similarities than differences in PGD symptoms between 255 men (mean age: 71.7 years) and 854 women (mean age: 69.7), with only differences in the relative importance of shock and greater strength of the female network (38). A study that focused on gender differences in grief trajectories showed varying symptom development between men and women (41). Men experienced PGD symptoms as an acute, decreasing reaction, whereas women showed lower grief reactions two months after the loss, but symptom increase in the months thereafter. In the current study, because of the limited sample size we were unable to detect interactions between gender and being refugee.

The study results imply that PGD might prevail in a considerable portion of patients who lost a loved one and were referred for treatment for common mental disorders. In particular, PGD symptom severity was found to be higher in patients with anxiety disorder. This suggests that treatment of the PGD might be overlooked in patients referred with common mental disorders, and in particular in patients with anxiety disorder. Moreover, the prevalence of PGD as well as symptom severity was higher in refugees than in non-refugees in our sample. Interestingly, refugee status had a higher impact on PGD symptom severity in younger than in older participants, although overall we did not find higher mean age to be related to PGD diagnosis as in other studies (4). Clinicians working with young refugees should therefore take particular care to identify and treat PGD symptoms. More generally, a recommendation from this study for clinicians is to check whether their patient has lost a loved one and if so, to carefully evaluate the presence or absence of PGD and to apply appropriate treatment.

Although this study has focused on the diagnosis of PGD and comorbidity with common mental disorders, the results imply that enhancing our knowledge on coping with bereavement and grief should be prioritized. More research on the treatment of PGD is required.

The current pilot study has some limitations. First, only patients with common mental disorders who lost a loved one were included. Therefore, the results only apply to these patients and cannot be generalized for other patient populations. Second, the relatively small sample size limits the power of the study to detect small effects. Since the study is cross-sectional, causal interpretations are not permitted. Research on the prevalence of PGD in larger samples is recommended. Third, a number of patients did not want to participate, because they did not want to talk about the loss of a loved one. This may be an indication that they are afraid to be confronted with their emotions regarding that loss and that they may suffer from PGD symptoms and that the prevalence of PGD symptoms may even be higher than the study results. Fourth, PGD criteria for DSM-5-TR and ICD-11 differ in terms of the period after the loss. DSM-5-TR PGD criteria are one year after the loss of a loved one and ICD-11 criteria are six months after the loss of a loved one. In this study, we included individuals who had lost a loved one a year and longer prior to the study, which excludes individuals who had lost a loved one between six months and a year prior to the study, which may jeopardize the perfect fit between DSM-5-TR and ICD-11 outcomes.

## Conclusion

In this sample of patients with common mental disorders who lost a loved one, one third met criteria for prolonged grief disorder (PGD) according to both criteria of ICD-11 and of DSM-5-TR. Patients with a refugee background showed two to three times higher chance of PGD than non-refugees, with women and patients with an anxiety disorder showing more severe PGD symptoms. The results of this study underscore the need for clinicians to screen for PGD in patients with common mental disorders, especially in refugee patients, and to treat them accordingly.

## Data Availability

The datasets presented in this study can be found in online repositories. The names of the repository/repositories and accession number(s) can be found below: https://osf.io/hmz9d/files/gmscx.
